# Supplementary material for: What determines the performance of low-carbon cities in China? Analysis of the grouping based on the technology—Organization—Environment framework
Source: PLoS One. 2023 Aug 15;18(8):e0289160. doi: 10.1371/journal.pone.0289160 (PMC10427000; doi:10.1371/journal.pone.0289160)
Supplement: S1 File — (DOCX) [file pone.0289160.s002.docx]

# Supporting Material 1-fsQCA's mathematical approach

For fuzzy sets, cases have a set affiliation ranging from 0.0 to 1.0, i.e., partial affiliation. A fuzzy subset relationship exists when the affiliation of a case in one set is consistently less than or equal to its affiliation in another set (Ragin, 2008). When plotted as a graph, fuzzy subset relations are triangular. For example, given a condition X and an outcome Y. In the graph, all values of X are less than or equal to their corresponding Y values. Therefore it can be determined that X is a subset of Y (sufficient condition).


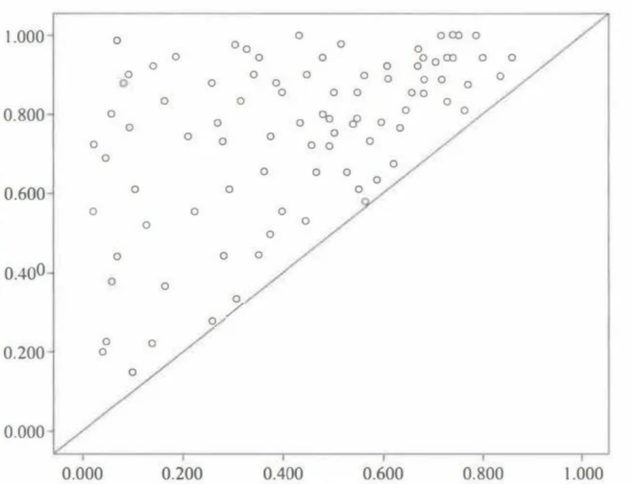


Figure source: Ragin, C. C. (2008). Redesigning social inquiry: Fuzzy sets and beyond: University of Chicago Press.

In the fuzzy set, the consistency of sufficient conditions is derived from a set of formulas, where the consistency of X (the subset of condition variables) as a subset of Y (the subset of outcome variables) is the proportion of their intersection to X.

$$Consistency(X_{i}\leq Y_{i})=\sum[\min(X_{i},Y_{i})]/\sum(X_{i})(1)$$

The formula for the degree of coverage is given by:

$$Coverage(X_{i}\leq Y_{i})=\sum[\min(X_{i},Y_{i})]/\sum(Y_{i})(2)$$

Necessary condition analysis examines the antecedent condition X as a superset of the outcome Y, in other words, the outcome Y as a subset of the condition X. The consistency of the necessary conditions is calculated by the formula:

$$Consistency(Y_{i}\leq X_{i})=\sum[\min(X_{i},Y_{i})]/\sum(Y_{i})(3)$$

The coverage degree is calculated as:

$$Coverage(Y_{i}\leq X_{i})=\sum[\min(X_{i},Y_{i})]/\sum(X_{i})(4)$$
