# Supplementary material for: What determines the performance of low-carbon cities in China? Analysis of the grouping based on the technology—Organization—Environment framework
Source: PLoS One. 2023 Aug 15;18(8):e0289160. doi: 10.1371/journal.pone.0289160 (PMC10427000; doi:10.1371/journal.pone.0289160)
Supplement: S2 File — (DOCX) [file pone.0289160.s003.docx]

# Supporting Material 2-Construction of indicators

**1.Outcome Variables - Low Carbon City Performance Assessment Indicators**

**1.1Methodology for selecting indicators**

The interaction of the economy, society, and environment - a concept known as the triple bottom line (TBL; Elkington, 1997) - has been widely adopted and long recognized in practice and research. Bocken et al. (2014), Joyce and Paquin (2016), Govindan et al. (2013), and Garcia et al. (2016) have all emphasized the significance of TBL.

In the context of China's low-carbon pilot cities, four aspects - carbon emission index, energy structure index, energy consumption index, and development quality index - can be effectively grouped into the TBL framework.

First, the carbon emission index can be seen as an environmental indicator within the TBL framework. It measures the amount of greenhouse gases emitted by a low-carbon pilot city and directly impacts the quality of the environment. By reducing carbon emissions, low-carbon pilot cities can contribute to improving air quality and mitigating the negative impacts of climate change. Second, the energy mix index and energy consumption index can be seen as economic indicators within the TBL framework. These indicators reflect the efficiency of energy use and the degree of dependence on non-renewable resources. Improving energy efficiency and transitioning to renewable energy sources will not only reduce costs but also drive economic growth and create new jobs in low-carbon pilot cities. Third, the Development Quality Index can be seen as a social indicator within the TBL framework. It measures the quality of life of residents and includes factors such as GDP per capita, Ratio of good air quality days in cities, and Greening coverage rate of urban built-up areas. By improving the overall quality of development, low-carbon pilot cities can improve the welfare of their residents and create more sustainable cities. Therefore, measuring the performance of China's low-carbon pilot cities in these four aspects is not only a key indicator to consider for China's low-carbon pilot work but also a reasonable and strong theoretical requirement for the TBL framework.

**1.2General description of the Low Carbon City Assessment System**

Based on this, this paper refers to the estimated ranking of each low-carbon pilot city in the performance score derived from the comprehensive report of the Research on China's Long-Term Low-Carbon Development Strategy and Transformation Path by the Institute of Climate Change and Sustainable Development of Tsinghua University and the Carbon Neutral Group under the Southern Finance and Economics All Media Group and other comprehensive measurements as the measurement index of the outcome variable (as shown in the figure below). The assessment index system takes the Net Zero Carbon City Index as the primary indicator, with four secondary indicators of carbon emission index, energy structure index, energy consumption index, and development quality index, and 33 tertiary indicators under it. The data sources of this report are from the Guangdong-Hong Kong-Macao Greater Bay Area (Guangdong) Financial Data Center; the Statistical Yearbook of each city in 2021; the Environmental Bulletin of each city in 2021; the data of the 7th National Population Census; the data of National Geographic Science and Technology; the energy bureau of each city; the electricity data published by public sources, and so forth. Therefore, the Net Zero Carbon City Index score derived from this report can reflect the performance of low-carbon city construction.

**1.3 Interpretation of selected key indicators**

**1.3.1Carbon Emission Index**

It mainly measures the current status of carbon emissions in cities and their reduction effectiveness. It focuses on carbon emissions from the power generation side and electricity consumption side of cities; total carbon emissions and intensity of cities and their changes. The report is based on the electricity data published by local energy bureaus and local statistical yearbooks, combined with carbon emission coefficients to calculate the carbon emissions and intensity of each city by formula. The report mainly judges the effectiveness of carbon reduction of cities through the change of total carbon emissions, and also selects carbon emissions per unit of GDP to measure the relationship between economic growth and carbon emissions growth of cities. China's energy endowment is coal-rich, oil-poor and gas-poor, and the current electricity supply is dominated by thermal power, which consumes a large amount of coal and is the main source of carbon dioxide emissions in China's energy production. The report evaluates the carbon emission level of urban power systems through carbon emissions on the power generation side and the electricity consumption side, aiming to encourage cities to change their energy structure, build a new power system with new energy as the main source, and reduce carbon emissions on the power generation side and the electricity consumption side.

**1.3.2Energy Structure Index**

The Energy Structure Index measures the relationship between the various types of energy sources at the power generation end of a city and their changes. Nearly 90% of China's carbon emissions come from the energy sector, and achieving the "double carbon" target requires an accelerated transformation of the energy mix. The report takes the proportion of thermal power generation in cities and its changes to evaluate the reduction of fossil energy use in cities. The "Action Plan to Reach the Carbon Peak by 2030" proposes to comprehensively promote the large-scale development and high-quality development of wind and solar power, insist on both centralized and distributed power generation, and accelerate the construction of wind and photovoltaic power generation bases. In the process of increasing the proportion of non-fossil energy, the development of wind power and photovoltaic power generation is the key. Therefore, the report takes the proportion of wind power and photovoltaic power generation and its changes as an important basis for assessing the transformation of the city's energy structure.

**1.3.3Energy Consumption Index**

The Energy Consumption Index measures the dependence of cities on energy consumption for economic development, and cities moving toward net zero carbon require more efficient energy use and lower energy consumption. The report examines the energy consumption level of cities from the perspective of energy consumption and electricity consumption.

Energy consumption intensity, i.e., the energy consumed per unit of GDP, reflects the city's dependence on energy for economic development and the efficiency of energy use on the one hand, and the city's technological level and production efficiency on the other. In this report, energy consumption per unit of GDP and energy consumption per capita and their changes are selected to measure the energy consumption level and energy saving and consumption reduction status of cities.

In 2020, China's coal consumption has decreased to 56.8% of total energy consumption from 72.4% in 2005, but coal consumption is still one of the main causes of CO2 emissions. Building a new energy system requires a gradual shift away from dependence on coal-based fossil energy. The report uses the proportion of coal consumption to energy consumption and its change to measure the effectiveness of urban energy transition from one side.

**1.3.4Quality of Development Index**

The development quality index mainly measures the level of economic development of cities, the economic support ability of net-zero carbon city construction and the foundation of net-zero carbon development of cities. Economic strength is the most direct reflection of a city's development capacity, and the stronger a city's economic strength, the more it has the need and ability to make systemic change adjustments. The environmental Kuznets curve suggests that environmental quality begins to degrade as income increases, and improves as income increases after income level rises to a certain level, i.e., environmental quality and income have an inverted U-shaped relationship. GDP per capita reflects the level of economic output of a city, and the report takes GDP per capita as an important basis for measuring the quality of urban economic development. Urbanization is a reflection of the degree of urban development and an important symbol of national modernization. Some studies believe that the urbanization rate of China's resident population is expected to approach or reach a peak in 2030-2035 and enter a relatively stable state. China proposes that CO2 emissions strive to reach a peak by 2030 and strive to achieve carbon neutrality by 2060. As we can see, the new urbanization and the "dual carbon" target are not only driving hand in hand but also cross-fertilizing and promoting each other in the historical process.

**1.4Data Processing**

Because of the different units of measure and orders of magnitude of each indicator, the data need to be standardized, and this study mainly uses the international standard frontier distance method to obtain the standardized scores of each indicator:

In case of positive indicators, the calculation formula:

$$Y_{ij}=\frac{x_{ij}-\min(x_{ij})}{\max(x_{ij})-\min(x_{ij})}*100(1)$$

In case of negative indicators, the calculation formula:

$$Y_{ij}=\frac{\max(x_{ij})-x_{ij}}{\max(x_{ij})-\min(x_{ij})}*100(2)$$

**1.5Data source**

Guangdong-Hong Kong-Macao Greater Bay Area (Guangdong) Financial Data Center; Statistical Yearbook of each city in 2021; Environmental Bulletin of each city in 2021; data of the 7th National Population Census; data of national and local science and technology; energy bureaus of each city; electricity data published by public sources, etc.

**Table 1.Low carbon city assessment index system**

| **Level 1 Indicators** | **Level 2 Indicators** | **Level 3 Indicators** |
| --- | --- | --- |
| **Net-Zero Carbon Cities Index（100%）** | **Carbon Emission Index（15%）** | Electricity carbon emissions from the use side（3%） |
|  |  | Electricity carbon emissions from the generation side（3%） |
|  |  | Total Carbon Emissions Change（3%） |
|  |  | Carbon emissions per unit GDP（3%） |
|  |  | Change in carbon emissions per unit GDP（3%） |
|  | **Energy Structure Index（18%）** | Proportion of coalfired power to total electricity（3%） |
|  |  | Change in the share of coalfired power（3%） |
|  |  | Proportion of photovoltaic power generation to total power generation（3%） |
|  |  | Change in the share of photovoltaic power generation（3%） |
|  |  | Proportion of wind power generation to total power generation（3%） |
|  |  | Change in the share of wind power generation（3%） |
|  | **Energy Consumption Index（36%）** | Change in total energy consumption（3%） |
|  |  | Energy consumption per unit GDP（3%） |
|  |  | Change in energy consumption per unit GDP（3%） |
|  |  | Energy consumption per capita（3%） |
|  |  | Change in energy consumption per capita（3%） |
|  |  | Change in total electricity consumption（3%） |
|  |  | Electricity consumption per unit GDP（3%） |
|  |  | Change in electricity consumption per unit GDP（3%） |
|  |  | Electricity consumption per capita（3%） |
|  |  | Change in electricity consumption per capita（3%） |
|  |  | Coal consumption as a proportion of energy consumption（3%） |
|  |  | Change in the share of coal consumption in energy consumption（3%） |
|  | **Quality of Development Index（31%）** | Total GDP（3%） |
|  |  | GDP per capita（3%） |
|  |  | GDP per capita growth rate（3%） |
|  |  | Rate of urbanization（4%） |
|  |  | GDP per unit area（3%） |
|  |  | Ratio of good air quality days in cities（3%） |
|  |  | General industrial solid waste disposal rate（3%） |
|  |  | Greening coverage rate of urban built-up areas（3%） |
|  |  | Carbon sequestration per unit area（3%） |
|  |  | Carbon Sequestration per capita（3%） |

2.条件变量——低碳城市绩效评估指标

**2.Technology -Digital Economy Development Level**

**2.1Methodology for selecting indicators**

The Technical Conditions:digital economy development level is a combined weight score from extensive data development and Internet development status. First, according to the three indexes used in Lian Yuming's (2020) "China's Big Data Development Report" to measure the Big Data development index of each region, the governmental use index, commercial use index, and civil use index, in order to measure the development level of Big Data in each region more comprehensively, this paper selects the comprehensive index to measure the overall level of Big Data development in each region. Second, according to the 2022 China Internet Development Status Statistical Report^⑦^.The proportion of regional names to the total number of domain names and the proportion of IPv4 to the total number of IPv4 in the country for each region used in the report are used as indicators, and the average value of them is measured comprehensively to arrive at the Internet development level of each region.

**2.2Data Processing**

**Table 2.Construction of digital economy development index indicators**

| Level 1 Indicators | Level 2 Indicators | Level 3 Indicators | Level 4 Indicators |
| --- | --- | --- | --- |
| Technology -Digital Economy Development Level（100%） | China Internet Development Status Index（20%） | The proportion of regional names to the total number of domain name by region（10%） | The proportion of "CN" domain names to the total number of "CN" domain names in each province（5%） |
|  |  |  | The proportion of "China" domain names to the total number of "China" domain names（5%） |
|  |  | Proportion of IPv4 to total national IPv4 by region（10%） | Proportion of IPv4 to total national IPv4 by region（10%） |
|  | Total index of big data development（80%） | Attention and Support（10%） | Development attention "big data" hot（5%） |
|  |  |  | Policy strength (number of big data-related policy releases)（5%） |
|  |  | Pilot Demonstration（10%） | Pilot innovation (big data comprehensive test area construction)（10%） |
|  |  | E-Government（10%） | Online government (online service level)（5%） |
|  |  |  | Data openness (level of data openness)（5%） |
|  |  | Development Support（20%） | Human resource base (average number of employees in the electronics and communications equipment manufacturing industry)（4%） |
|  |  |  | Scale of related industries (software and IT service revenue as a percentage of GDP)（4%） |
|  |  |  | Application benefit degree (the proportion of Internet users of APP applications to the national Internet users)（4%） |
|  |  |  | Business penetration (number of websites per 100 companies)（4%） |
|  |  |  | Network security (number of network security pilot demonstration projects in the telecommunications and Internet industries)（4%） |
|  |  | Digital Foundation（10%） | Terminal penetration (cell phone penetration rate)（5%） |
|  |  |  | Network base (number of mobile Internet access traffic per capita)（5%） |
|  |  | Digital Convenience（10%） | Service Accessibility (Smart City Impact)（10%） |
|  |  | Digital Capabilities（10%） | Digital skills (average years of education)（5%） |
|  |  |  | Consumption capacity (the proportion of residents' transportation and communication expenditures to total consumption expenditures)（5%） |

**3.Technology-Low carbon economy development level**

**3.1Methodology for selecting indicators**

The Technical Conditions:Level of Low Carbon Economic Development is measured based on the relevant indicators of the "Evaluation Report on the Business Environment of China's Large and Medium-sized Cities in 2020," jointly published by the Guangdong-Hong Kong-Macao Greater Bay Area Research Institute and the 21st Century Institute of Economic Research. The report uses data from various statistical yearbooks and administrative records, as well as data from several big data companies such as the Qixinbao, to measure 296 cities at all prefectural levels as well as above prefectural levels, involving online indicators for the whole life cycle of enterprises, investment attractiveness, and high-quality development, including business start-up, market supervision, innovation and entrepreneurial activity, ecological environment, science and technology innovation, and other indicators—relevant indicators from the China City Statistical Yearbook and the China Environment Statistical Yearbook.

**3.2Data Processing**

**Table 3: Construction of low carbon economic development index indicators**

| Level 1 Indicators | Level 2 Indicators | Level 3 Indicators |
| --- | --- | --- |
| Low carbon economy development level（100%） | Green production motivation（27%） | Relative ranking of the number of high-tech enterprises among national cities（9%） |
|  |  | Growth rate of the number of high and new technology enterprises（9%） |
|  |  | Total Factor Productivity（9%） |
|  | Attraction of human resources（25%） | Growth rate of resident population（5%） |
|  |  | Population increment（5%） |
|  |  | Annual payroll index（5%） |
|  |  | Number of secondary schools and above（5%） |
|  |  | Number of fresh graduates from secondary schools and above（5%） |
|  | Investment attractiveness（8%） | Amount of foreign investment utilized（8%） |
|  | Innovation Activity（20%） | Number of market entities created per 10,000 people（10%） |
|  |  | Number of enterprises created per 10,000 people indicator（10%） |
|  | Market Regulation（20%） | Index of revoked enterprises（10%） |
|  |  | Cancellation of business index（10%） |

**4.Organizational-Level of importance of the parent organization**

**4.1Methodology for selecting indicators**

The Organizational Conditions:The are important for higher-level organizations. According to the authors, about 15 provinces have formally issued the Implementation Plan for Carbon Peaking by the end of 2022, including Beijing, Shanghai, Tianjin, Jiangsu, Hunan, and other provinces. The authors combed through the formally issued implementation plans of each province's carbon peaking, summarized the specific objectives and task measures, and measured indicators. The figure below shows.

**4.2Data Processing**

**Table 4: Indicator measures related to the Carbon Dump Implementation Program**

| Level 1 Indicators | Level 2 Indicators |
| --- | --- |
| Organizational-Level of importance of the parent organization | Whether to introduce documents on the implementation of carbon peaking program（25%） |
|  | When the document was introduced（25%） |
|  | The proportion of non-fossil energy consumption mentioned in the document to reach by 2025（10%） |
|  | The document mentions energy consumption per unit of GDP and CO2 emissions per unit of GDP by 2025（10%） |
|  | The document mentions the proportion of non-fossil energy consumption by 2030（10%） |
|  | The document mentions the rate of reduction of CO2 emissions per unit of regional GDP by 2030（10%） |
|  | Is there any mention in the document of important initiatives to be introduced（10%） |

**5.Level of government transparency and interaction**

**5.1Methodology for selecting indicators**

The Organizational Conditions:The degree of government transparency and interaction is derived from the combined weighted scores of the Government Transparency Index and the Government Microblogging Competitiveness Index. One is a comprehensive measurement based on the Chinese Government Transparency Index Report released by the Institute of Law, Chinese Academy of Social Sciences in 2020. The level of government transparency and government information disclosure in each region is obtained from the total score of government information disclosure by averaging five indicators: openness in decision-making (20%), openness in management services (25%), openness in implementation and results (15%), openness in critical areas of information (20%), and policy interpretation and response to concerns (20%). The second is based on the "2020 Government Affairs Index Microblog Influence Report" released by the People's Daily Online Public Opinion Data Center and the Microblog Data Center, which aims to assess the comprehensive application ability and application effect of new media in each region. The sub-indicators communication, service, and interaction power are weighted and standardized to determine the "government affairs microblog competitiveness index's overall score."

**5.2Data Processing**

**Table 5. Construction of indicators for the degree of government transparency and interaction**

| Level 1 Indicators | Level 2 Indicators | Level 3 Indicators |
| --- | --- | --- |
| Level of government transparency and interaction | Openness in decision making (20%) | Pre-disclosure of major decisions (10%) |
|  |  | Suggestions and proposals for the results of the public (10%) |
|  | Management service disclosure (20%) | Power list public (5%) |
|  |  | Information disclosure of government services (5%) |
|  |  | "Double random" supervision information disclosure (5%) |
|  |  | Administrative punishment information disclosure (5%) |
|  | Implementation and results disclosure (15%) | Disclosure of audit results (5%) |
|  |  | Annual report on the construction of the rule of law government (5%) |
|  |  | Government Work Report (5%) |
|  | Information disclosure in key areas (20%) | Normative document disclosure (7%) |
|  |  | Financial budget disclosure (7%) |
|  |  | Urban water environment quality ranking (6%) |
|  | Policy interpretation and response to concerns (15%) | Policy Interpretation (10%) |
|  |  | Responding to concerns (5%) |
|  | Competitiveness index of official government microblogs (10%) | Dissemination power indicators (4%) |
|  |  | Service power indicators (3%) |
|  |  | Interaction power indicators (3%) |

**6.Environment - Green Development Level**

**6.1Methodology for selecting indicators**

The Environmental Conditions: Green Development Level is a combined weighted score from the 2020 China Large and Medium Cities Business Environment Evaluation Report and China Regional Marketability Index data. First, it is measured through the indicators of the "2020 Evaluation Report on the Business Environment of Large and Medium-sized Cities in China" jointly published by the Guangdong-Hong Kong-Macao Greater Bay Area Research Institute and the 21st Century Institute of Economic Research. The report uses data from various statistical yearbooks and administrative records, as well as data from several big data companies such as Qixinbao, to measure 296 cities at all prefectural levels and above, involving online indicators for the whole life cycle of enterprises, investment attractiveness, and high-quality development, including business start-up, market supervision, innovation and entrepreneurial activity, ecological environment, science and technology innovation, and other indicators. Second, the data of the China Regional Marketization Index, which measures the regional marketization level of each province and city according to Fan Gang's "Marketization Index Report" (2021).

**6.2Data Processing**

**Table 6. Construction of indicators of green development level**

| Level 1 Indicators | Level 2 Indicators | Level 3 Indicators | Level 4 Indicators |
| --- | --- | --- | --- |
| Environment - Green Development Level（100%） | Urban Infrastructure Development Index（35%） | Road network density（6%） | Density of road network in built-up areas（3%） |
|  |  |  | Road area per capita（3%） |
|  |  | Internet Level（6%） | Mobile Internet Mobile Number（3%） |
|  |  |  | Number of broadband households（3%） |
|  |  | Road freight（4%） | Road freight volume（4%） |
|  |  | Waterborne freight（4%） | Waterway freight volume（4%） |
|  |  | Civil Aviation Transportation（3%） | Civil aviation transport volume（3%） |
|  |  | Gas supply（3%） | Gas supply（3%） |
|  |  | Water Supply（3%） | Water Supply（3%） |
|  |  | Subways（3%） | Length of subway（3%） |
|  |  | Cabs（3%） | Number of cabs（3%） |
|  | Total Market Index（25%） | Resident population（4%） | Resident population（4%） |
|  |  | Regional Gross Domestic Product（3%） | Regional Gross Domestic Product（3%） |
|  |  | Industrial upgrading（3%） | Share of tertiary sector in regional GDP（3%） |
|  |  | Total retail sales of social consumer goods（3%） | Total retail sales of social consumer goods（3%） |
|  |  | General budget revenue（3%） | General budget revenue（3%） |
|  |  | Import and export volume（3%） | Import and export volume（3%） |
|  |  | Loan amount（3%） | Loan amount（3%） |
|  |  | Disposable income per capita（3%） | Disposable income per capita（3%） |
|  | Business Cost Index（12%） | Utilities Cost Index（4%） | Comprehensive cost of utilities（4%） |
|  |  | Wage costs（4%） | Wage costs（4%） |
|  |  | Land Cost Measurement（4%） | House price (third party end of 2019 data) income ratio measurement（4%） |
|  | Social Service Index（28%） | Financing（4%） | Number of financing companies（4%） |
|  |  | Technology（4%） | Amount of R&D investment in science and technology（4%） |
|  |  | Medical（4%） | Number of medical beds per 1,000 people（4%） |
|  |  | Retirement（4%） | Number of urban pension participants（4%） |
|  |  | Education（4%） | Number of secondary schools and above（4%） |
|  |  | Human Resources（4%） | Total number of college students（4%） |
|  |  | R&D Service（4%） | Number of patent applications per 10,000 people（4%） |

**7.Environmental-ecological resource endowment status**

**7.1Methodology for selecting indicators**

The Environmental Conditions:Ecological resource endowment status. The relevant data are measured and derived from the relevant indicators in the China Urban Statistical Yearbook and the China Environmental Statistical Yearbook, which include six secondary and 48 tertiary indicators, as shown in the figure below.

**7.2Data Processing**

**Table 7. Construction of indicators of ecological resource endowment status**

| Level 1 Indicators | Level 2 Indicators | Level 3 Indicators |
| --- | --- | --- |
| Environmental-ecological resource endowment status(100%) | Atmosphere(20%) | Annual average concentration of fine particulate matter(2%) |
|  |  | Sulfur Dioxide(3%) |
|  |  | Annual average sulfur dioxide concentration (µg/m³)(2%) |
|  |  | Annual average concentration of nitrogen dioxide (µg/m³)(2%) |
|  |  | Annual average concentration of respirable particulate matter (PM10) (µg/m3)(2%) |
|  |  | 95th percentile daily average carbon monoxide concentration (mg/m³)(2%) |
|  |  | Ozone (O3) maximum 8-hour 90th percentile concentration (µg/m³)(2%) |
|  |  | Annual average concentration of fine particulate matter (PM2.5) (µg/m³)(2%) |
|  |  | Number of days when air quality is best attained and better than secondary (days)(3%) |
|  | Greenfield(12%) | Green space coverage of built-up areas(2%) |
|  |  | Harmless treatment rate of domestic waste(2%) |
|  |  | Forest area (million hectares)(2%) |
|  |  | Forest cover (2%) |
|  |  | Total standing wood accumulation (billion cubic meters)(2%) |
|  |  | Forest stock (billion cubic meters)(2%) |
|  | Water(12%) | Water quality of surface water(2%) |
|  |  | Unit area of water resources(2%) |
|  |  | Total water resources(2%) |
|  |  | Surface Water Resources(2%) |
|  |  | Groundwater Resources(2%) |
|  |  | Per capita water resources ( m³/person )(2%) |
|  | Temperatures(9%) | Annual average temperature (Celsius) year-on-year increase (decrease) rate(3%) |
|  |  | Annual extreme maximum temperature (degrees Celsius) year-on-year increase (decrease) rate(3%) |
|  |  | Annual extreme minimum temperature (degrees Celsius) year-on-year increase (decrease) rate(3%) |
|  | Energy(22%) | City gas penetration rate(2%) |
|  |  | Total primary energy production (million tons of standard coal)(2%) |
|  |  | Energy production per capita (kg standard coal/person)(2%) |
|  |  | Total energy consumption (million tons of standard coal)(2%) |
|  |  | Per capita energy consumption (kg standard coal/person)(2%) |
|  |  | Energy production elasticity factor(2%) |
|  |  | Energy consumption elasticity coefficient(2%) |
|  |  | Electricity production elasticity factor(2%) |
|  |  | Electricity consumption elasticity coefficient(2%) |
|  |  | Energy consumption of 10,000 yuan GDP (tons of standard coal / 10,000 yuan)(2%) |
|  |  | Total natural gas supply(million cubic meters)(2%) |
|  | Pollutant Emissions(25%) | Chemical oxygen demand emissions (million tons)(2%) |
|  |  | Ammonia nitrogen emissions (million tons)(2%) |
|  |  | Sulfur dioxide emissions (million tons)(2%) |
|  |  | Nitrogen oxide emissions (million tons)(2%) |
|  |  | General industrial solid waste comprehensive utilization volume (million tons)(2%) |
|  |  | Municipal domestic waste removal volume (million tons)(2%) |
|  |  | Industrial wastewater discharge data per unit area(2%) |
|  |  | Municipal sewage discharge (million cubic meters)(2%) |
|  |  | Industrial wastewater discharge(2%) |
|  |  | Agricultural wastewater discharge(2%) |
|  |  | Domestic wastewater discharge(2%) |
|  |  | Number of centralized pollution control facilities for wastewater(2%) |
|  |  | Urban sewage treatment rate(1%) |
