# Supplementary material for: What determines the performance of low-carbon cities in China? Analysis of the grouping based on the technology—Organization—Environment framework
Source: PLoS One. 2023 Aug 15;18(8):e0289160. doi: 10.1371/journal.pone.0289160 (PMC10427000; doi:10.1371/journal.pone.0289160)
Supplement: Attachment — Submitted filename: Review (2).docx [file pone.0289160.s004.docx]

The authors explore the linkage effects of technology, organization and environmental conditions on the development performance of low-carbon cities and their path choices by using the fuzzy set qualitative comparative analysis (fsQCA) method and proposes two types of solutions to promote the performance of low-carbon cities, namely, the standard explanation type of technology, organization and environmental elements and the standard explanation type of organization and environment in the matching model. The topic is quite novel, but the argumentation process is not rigorous enough, there are still some areas for improvement in the paper.

1. Please add some appropriate management implications to the abstract.

2. The purpose and relevance of the article should be more accurately expressed. This is more obvious in the introduction and model. In the model analysis, why use the fsQCA method, compared with the existing research, choose this method. Further analysis is necessary to better prepare and discuss the results. What is the model formulation specificity and excellence used?

This needs further improvement.

3. In 3.2.1 Resulting variables, ‘The results of this paper are concerned with the construction performance of low-carbon pilot cities in China. According to the three batches of low-carbon cities piloted by the China Development and Reform Commission from July 2010 to the present, the positioning of China's low-carbon pilot cities mainly includes four aspects:carbon emission index, energy structure index, energy consumption index, and development quality index. Therefore, measuring its construction performance should also start from these four aspects. ’Please add some theory to support your idea.

4. Figure 2. Low carbon pilot city development performance map. The map is not so clear and it should be presented in English please.

4. Results: It is understood that the model of the analysis has been done properly. An in-depth discussion should be given to support the purpose of the research.

5.Please make a discussion in which the Authors would compare their results to those from other publications.

6. The conclusions should be more precise.
