# Supplementary material for: What determines the performance of low-carbon cities in China? Analysis of the grouping based on the technology—Organization—Environment framework
Source: PLoS One. 2023 Aug 15;18(8):e0289160. doi: 10.1371/journal.pone.0289160 (PMC10427000; doi:10.1371/journal.pone.0289160)
Supplement: Attachment — Submitted filename: PONE-D-23-08832_reviewer_Comments.docx [file pone.0289160.s005.docx]

This paper examines the performance of low-carbon cities in China using fuzzy set qualitative comparative analysis (fsQCA). It's an interesting topic, but there is a lot of room for improvement.

1. The author should avoid using abbreviations in the title.

2. The author needs to further conclude the research problem and research framework to clearly tell the reader what you have done in this paper.

3. The policy implications are also unclear. The author mentions that "cities choose appropriate pathways and targeted measures according to their own characteristics and resource endowments...". However, it is easy to say that what to do is what really matters. Moreover, there seems to be no link between the results and the policy implications.

4. The literature review should be in a dependent part or with in the Introduction, rather the research framework.

5. The author should conclude the previous studies instead of listing the studies and their results one by one. These following articles give an example on how to carry out a literature review.

Meng, C. et al. (2020). Sustainable urban development: An examination of literature evolution on urban carrying capacity in the Chinese context. Journal of Cleaner Production, 277, 122802.

Meng, C.et al. (2023). The static and dynamic carbon emission efficiency of transport industry in China. Energy, 127297.

6. Non-researchers used the fsQCA method to explore the performance of low carbon cities in the literature review. Are you using this methodology for the first time to investigate the performance of low carbon cities? If so, what consumption or research are you doing to ensure the application of this method?

7. The mathematics of fsQCA is necessary to present in the method.

8. What method do you use to select the indicators and how do you weight each indicator? The author should provide more explanation.

9. The words in Figure 2 should be translated into English.

10. The words in Figures 3 to 8 are too small.

11. The author should explain the reasons for the results, not just describe them.

12. Lack of communication with previous studies discussed in the literature review section. These articles are also focus on the performance of low-carbon cities construction.

Du, X., et al. (2023). An improved approach for measuring the efficiency of low carbon city practice in China. *Energy*, 126678.

Shen, L., et al. (2021). Temporal-spatial evolution analysis on low carbon city performance in the context of China. *Environmental Impact Assessment Review*, *90*, 106626.

Wang, C.et al. (2013). Policies and practices of low carbon city development in China. *Energy & Environment*, *24*(7-8), 1347-1372.

13. The policy implications are also missing.

14. The whole article has poor readability. There are many grammatical errors, such as the first paragraph in the Introduction "in 2020", which should be "In 2020".
