# Supplementary material for: What determines the performance of low-carbon cities in China? Analysis of the grouping based on the technology—Organization—Environment framework
Source: PLoS One. 2023 Aug 15;18(8):e0289160. doi: 10.1371/journal.pone.0289160 (PMC10427000; doi:10.1371/journal.pone.0289160)
Supplement: Attachment — Submitted filename: 0614Second Modification-Response to Reviewers.docx [file pone.0289160.s006.docx]

Dear Editor

I am very glad to receive your revision letter, which is an affirmation and encouragement of my last revision result. I have carefully read the corrections you made in your email, and we have incorporated each of your comments and revised them in the corresponding section of the article.

**1.Suggestion：**Please note that PLOS ONE has specific guidelines on code sharing for submissions in which author-generated code underpins the findings in the manuscript. In these cases, all author-generated code must be made available without restrictions upon publication of the work. Please review our guidelines at https://journals.plos.org/plosone/s/materials-and-software-sharing#loc-sharing-code and ensure that your code is shared in a way that follows best practice and facilitates reproducibility and reuse.Please provide your response in the "Author Comments" section.

**Response:**I have carefully reviewed the relevant guidelines ([https://journals.plos.org/plosone/s/materials-and-software-sharing#loc-sharing-code](https://journals.plos.org/plosone/s/materials-and-software-sharing#loc-sharing-code)). This paper falls under the category of "Exceptions to sharing materials," specifically referring to situations where "Materials should not be shared if they compromise the privacy or confidentiality of human research subjects, and authors should include a statement in their Materials and Methods discussing any restrictions on availability or use."

The data utilized in this study were obtained from several reputable publications, namely the China 21st Century Finance and Economics Net Zero Carbon Cities Report, China City Statistical Yearbook, Statistical Yearbook of Chinese Provinces, China Big Data Development Report, China Internet Development Status Statistical Report, China Business Environment Evaluation Report for Large and Medium-sized Cities in 2020, China Environment Statistical Yearbook, Carbon Peak Implementation Plans of Chinese Provinces, Chinese Government Transparency Index Report (China Blue Book Series), 2020 Microblog Influence Report of Government Affairs Index (published by People's Daily), and Marketization Index Report (China Blue Book Series) edited by Fan Gang.

Due to copyright restrictions, the aforementioned data requires payment for access and is limited to paid scholarly research. Therefore, we are unable to provide it. Researchers who wish to examine the aforementioned publications and data can acquire them through their own purchase.

**2.Suggestion** .We note that your author list was updated during the revision process. Please complete our Authorship Change Form by following this link: http://journals.plos.org/plosone/s/file?id=13d0/plos-one-change-to-authorship-form.docx If you are adding or removing more than 2 authors, you can complete multiple forms and submit as many forms as needed to reflect the updates. Please return the form(s) as an attachment by emailing plosone@plos.org or by uploading it as a submission file labeled with the file type ‘Other’. Please note that if your manuscript is accepted, we will not be able to complete the publication process without the completed form.

**Response:**

The authors of our preliminary manuscript, submitted on March 24, are listed as follows: Quanling Cai∗, Mingxing Wang, Kaisheng Di, Dongli Li, Caiping Liu (∗E-mail addresses: [1022209089@tju.edu.cn](mailto:1022209089@tju.edu.cn)).

The authors of our major revision manuscript, submitted on May 23, are as follows: Weidong Chen☯, Quanling Cai☯∗, Kaisheng Di, Dongli Li, Caiping Liu, Mingxing Wang, Sichen Liu, Zhensheng Di, Qiumei Shi (☯These authors contributed equally to this work.* [1022209089@tju.edu.cn](mailto:1022209089@tju.edu.cn)).

Four new authors have been added to this paper:

Weidong Chen: Weidong Chen provided theoretical suggestions for the research design of this paper and offered corresponding support. Therefore, we include Weidong Chen as a co-author.

Sichen Liu: Sichen Liu assisted in the process of data review and provided related suggestions during the paper revision. For these reasons, we need to add Sichen Liu as a co-author of this paper.

Zhensheng Di: Zhensheng Di assisted in the process of data review and provided related suggestions during the paper revision. For these reasons, we need to add Zhensheng Di as a co-author of this paper.

Qiumei Shi: Qiumei Shi assisted in the process of data review and provided related suggestions during the paper revision. For these reasons, we need to add Qiumei Shi as a co-author of this paper.

The revised article, including the addition of two tables listing the four new authors, has been sent as an attachment to [plosone@plos.org](mailto:plosone@plos.org). Please label it as a submission under the "Other" file type.

**3.Suggestion**.Please ensure that you refer to Figures 1, 4, 5 and 12 in your text as, if accepted, production will need this reference to link the reader to the figure.

**Response:**We express our gratitude to the editor for their meticulousness and dedication in identifying this oversight. To ensure that all figures mentioned in the paper align appropriately, we have conducted a thorough analysis and comparison of Figures 1-12 in both the Revised Manuscript with Track Changes and the Manuscript.

**4.Suggestion**.Please ensure that you refer to Tables 2, 3, 5 and 9 in your text as, if accepted, production will need this reference to link the reader to the Table.

**Response:**We express our appreciation to the editor for their meticulousness and dedication in identifying this oversight. To ensure the consistent inclusion of Table 1-13 in both the Revised Manuscript with Track Changes and the Manuscript, we have conducted a thorough analysis and comparison of each table.

**5.Suggestion**.We note your current Data Availability statement is:

"No - some restrictions will apply"

"China Statistical Yearbook;China City Statistical Yearbook"

"Tick here if the URLs/accession numbers/DOIs will be available only after acceptance of the manuscript for publication so that we can ensure their inclusion before publication."

PLOS' data policy allows authors to submit their manuscript and include placeholder language in their Data Availability statement indicating that accession numbers and/or DOIs will be made publicly available after acceptance. The journal office will then contact authors prior to publication to ask for this information and will hold the paper until it is received.

In this case, providing private data access to reviewers and editors during the peer review process is acceptable. Many repositories permit private access for review purposes, and have policies for public release at publication. (https://journals.plos.org/plosone/s/data-availability#loc-data-policy-faqs)

Before we can proceed, please address the following prompts:

a.) Please confirm whether you intend to provide public access to your data at the provided accession numbers if your submission is accepted for publication.

b.) Otherwise, at this time, please upload the minimal anonymized dataset necessary to replicate your study findings to a stable, public repository and provide the accession numbers or DOIs necessary to access these data. For a list of recommended repositories, please see https://journals.plos.org/plosone/s/recommended-repositories. You also have the option of uploading the data as Supporting Information files, but we would recommend depositing data directly to a data repository if possible.

We will update your Data Availability statement on your behalf to reflect the information you provide.

**Response:**

The data presented in this paper have been sourced from official Chinese government documents as well as paid data and books. Due to these circumstances, we are unable to disclose the entirety of the data within this paper. However, we are able to provide access to a subset of the data, which can be disclosed and uploaded as supporting documents.

Researchers interested in accessing the data are kindly requested to contact the corresponding author of this paper via the provided email address ([1022209089@tju.edu.cn](mailto:1022209089@tju.edu.cn)). Upon contact, we will provide the necessary guidance to facilitate the purchase of the paid data and associated materials.
